# Supplementary material for: Rapid turnover of life-cycle-related genes in the brown algae
Source: Genome Biol. 2019 Feb 14;20:35. doi: 10.1186/s13059-019-1630-6 (PMC6374913; doi:10.1186/s13059-019-1630-6)
Supplement: Supplementary file 3 — Protein alignments, corresponding Gblocks html file and CDS sequences for the lineage conserved gametophyte-biased genes. (ZIP 2109 kb) [file 13059_2019_1630_MOESM3_ESM.zip › Con_GA_alignments/SJ12098.aa_ali.fasta-gb.htm]

SJ12098.aa.fasta.trans.aa\_ali.fasta


## Gblocks 0.91b Results

Processed file: **SJ12098.aa.fasta.trans.aa\_ali.fasta**  
Number of sequences: **2**  
Alignment assumed to be: **Protein**  
New number of positions: **383** (selected positions are underlined in blue)

```
                         10        20        30        40        50        60
                 =========+=========+=========+=========+=========+=========+
SJ12098          MHGKRQPATSGDILWGAARRLTASKFRATDGNEACGSVEKKILARNLDNVNKIWRAVALV
Mac.61824.1      ------------------------------------------------------------
                                                                             


                         70        80        90       100       110       120
                 =========+=========+=========+=========+=========+=========+
SJ12098          IESSLLSSAARGVRIESLGTFALDAKDRERFFLASEFVARHRLLKYDACTGGSLAGGSVN
Mac.61824.1      ------------------------------------------------------------
                                                                             


                        130       140       150       160       170       180
                 =========+=========+=========+=========+=========+=========+
SJ12098          TKLNISRVAAAAGSPRPEAERVLDAVLRSLRQCLASGRSVTLSFHPVAEFSCAPSRQSRM
Mac.61824.1      ------------------------------------------------------------
                                                                             


                        190       200       210       220       230       240
                 =========+=========+=========+=========+=========+=========+
SJ12098          RYLPGFRARKKAVAAAVTRGRVGVGRAKSKPTPWTAGSNNNRDTSAAVATAAAASAGESR
Mac.61824.1      ------------------------------------------------------------
                                                                             


                        250       260       270       280       290       300
                 =========+=========+=========+=========+=========+=========+
SJ12098          KTTRTTTTVANSRVDVRSPPVAGQFQARETAAAAVGTGRPLSASVSASETDRIVSDRPKG
Mac.61824.1      ------------------------------------------------------------
                                                                             


                        310       320       330       340       350       360
                 =========+=========+=========+=========+=========+=========+
SJ12098          SHGGAGSGALGANIDSGADGSTLRAAAESAVGAATTQPSSAWESPRTSRHNMENVARLTT
Mac.61824.1      ------------------------------------------------------------
                                                                             


                        370       380       390       400       410       420
                 =========+=========+=========+=========+=========+=========+
SJ12098          AYGSCASLGSEVFSGDPQHARCLKSFGPGDNGRKDAAAVYRCDGHGHLPEEEWLASLLRQ
Mac.61824.1      ------------------------------------------------------------
                                                                             


                        430       440       450       460       470       480
                 =========+=========+=========+=========+=========+=========+
SJ12098          QTIAQTGGEGLRRLTRTLRLTHADVRDRDGRLSGRSLLQALRDVGVALTSGELAEMASVF
Mac.61824.1      ------------------------------------------------------------
                                                                             


                        490       500       510       520       530       540
                 =========+=========+=========+=========+=========+=========+
SJ12098          RRQRDGRVSLHVLLAAIECDLCSTPSVEQTPELSPARVRGWREEERRGGSPCGIRRTRDS
Mac.61824.1      ------------------------------------------------------------
                                                                             


                        550       560       570       580       590       600
                 =========+=========+=========+=========+=========+=========+
SJ12098          KVRGGMPRQECSPTPSSGQRSDALSDPPCNTSKDPQDGAELLARGGERNEPQGRRDVEKR
Mac.61824.1      ------------------------------------------------------------
                                                                             


                        610       620       630       640       650       660
                 =========+=========+=========+=========+=========+=========+
SJ12098          YGDGSRQDKFRSRHGHGRPSSSPHDNIVDKTMGLQVGVPAMSQLPRECWHSSSGEGERKD
Mac.61824.1      ------------------------------------------------------------
                                                                             


                        670       680       690       700       710       720
                 =========+=========+=========+=========+=========+=========+
SJ12098          KASSEERAIAELATIVYHPPRSLEGLIHVLQASKVTEQPTIACAAMSARLCQLRPGLGRT
Mac.61824.1      ------------------------------------------------------------
                                                                             


                        730       740       750       760       770       780
                 =========+=========+=========+=========+=========+=========+
SJ12098          RAKELATAAACRRLDKKVCLAQLHALLERRFGRSGFTPTATATGRNIPTRPLASSSVGLS
Mac.61824.1      ------------------------------------------------------------
                                                                             


                        790       800       810       820       830       840
                 =========+=========+=========+=========+=========+=========+
SJ12098          CAAVERVRRNLLSGKGGLGGLKAALSKASASAEGSLTKEELRRALTIAGGSGCPPPTAKD
Mac.61824.1      ------------------------------------------------------------
                                                                             


                        850       860       870       880       890       900
                 =========+=========+=========+=========+=========+=========+
SJ12098          VDDLFTLFSCQTSAGVNGGRVEATTVLRSLRGDLSPARESVVRDVFRSLVNLSRSGHLVD
Mac.61824.1      ------------------------------------------------------------
                                                                             


                        910       920       930       940       950       960
                 =========+=========+=========+=========+=========+=========+
SJ12098          DVVGIPTDQGHGQIYSYTKRKVGMKRAMNDTVHVEDLKNAFCKGGGSGFSPVNGNAAPAC
Mac.61824.1      -------------------------------------------------SLVNGYTVPGC
                                                                  ###########


                        970       980       990      1000      1010      1020
                 =========+=========+=========+=========+=========+=========+
SJ12098          DRGNISTGGWFSGQVWPDDGSPRTSRSGAKMWGAGGKAANNGVTVEQFLEQYRDVSAGID
Mac.61824.1      DRSNSSVGGWFLGWLRPGDGSPRTSRSEGNTGGVGRKVDSNWVTVEQFLEHYRDVSAGID
                 ############################################################


                       1030      1040      1050      1060      1070      1080
                 =========+=========+=========+=========+=========+=========+
SJ12098          GDDAFEAFVRGSWSLRPLAHSEKNNDTIAASERGREIAVGAGRAGRTPPRTAPAWYGNNG
Mac.61824.1      GDDAFEAFVRGSWSMRPLAHPGQTNNILTASERGQKIDASAGGASRKP-RTAPARHGDGN
                 ################################################            


                       1090      1100      1110      1120      1130      1140
                 =========+=========+=========+=========+=========+=========+
SJ12098          TICNIIPDKTAQTSSRTVGVPLYARTGGTAPGRHHSIPRENNTGGKPAVGSPCVTSVVHQ
Mac.61824.1      HEGARSPDMIAQSIPRNVDVPLSARTGGTAPGRHHSFPREQKTAEAPAVGSPCSATFVHR
                       ######################################################


                       1150      1160      1170      1180      1190      1200
                 =========+=========+=========+=========+=========+=========+
SJ12098          ANYLSPVVSRMGELWRSTSMPPGAGQPGRKTHHTGATNDGSARKREGSPSRPVTAARRDA
Mac.61824.1      PNFSAPAVSRMRELPRAVSTPPEVRLPGRKQCHTGVTNARSVRGVEGSPVRPCTAARRDS
                 ############################################################


                       1210      1220      1230      1240      1250      1260
                 =========+=========+=========+=========+=========+=========+
SJ12098          DNTYGERSSARVGATTLQAKERMGVEDAHRRGRPATQRARWAPGDKQSGDGGGSRKYREE
Mac.61824.1      DDTYEEGSSAHG---ATKSKERRGIEGVHRCRRLATRRAWRVPGDEESGDGSGSHKYRQK
                 ##########         #########################################


                       1270      1280      1290      1300      1310      1320
                 =========+=========+=========+=========+=========+=========+
SJ12098          RVPQRQTQHEQGQGH-QAEELKGYRVE-------QQRAGTAGLGVTAIPAAAADLNVRQH
Mac.61824.1      GRPQRQTQHEQRRGQGQEKELRGYRVEPAAYEFEQQQAETVGLGVTAAPAAAADVNVRQQ
                 ##############  ###########       ##########################


                       1330      1340      1350      1360      1370      1380
                 =========+=========+=========+=========+=========+=========+
SJ12098          NQPPVDITTARSAEGCRQQPTTYIGLGKATRLYTGGGTTTAAFLLTGPRPPFACNESSED
Mac.61824.1      NQPPADTTTERVSEFQQD---------------------------------FERNERVAQ
                 ##############                                              


                       1390      1400      1410      1420      1430      1440
                 =========+=========+=========+=========+=========+=========+
SJ12098          TEEGSVQARKGGSERDNGLRSGKQIWSERGPTQPHATWPAAAHLPCPAAAVAAVVAVAAV
Mac.61824.1      NPKN----RRVLSTTNTETRTGK--------SEALVHGRGHAGRSVPFDRSAAAVRVQRV
                         ###############                  ###################


                       1450      1460      1470      1480      1490      1500
                 =========+=========+=========+=========+=========+=========+
SJ12098          AQQFSVFVKLVPFLSRVNHRREFAPTHQAVIGNCIIISTTAGNGHLNLIDMVHSRAPAAL
Mac.61824.1      FR---------------GHR----------------GRRCAG---------AEGRKRASQ
                                                                             


                       1510      1520      1530      1540      1550      1560
                 =========+=========+=========+=========+=========+=========+
SJ12098          NSYASEGEFPLFVATFYGHNTVVSKLLPLGAMQRIPRNSMCPLLVAVDKGFVIVERALIN
Mac.61824.1      R--ASEREAKMERERPHAASNRLSGRLPR---PKLPS----------T------------
                                                                             


                 
                 ===
SJ12098          GGG
Mac.61824.1      YGF
```

```
Parameters used
Minimum Number Of Sequences For A Conserved Position: 2
Minimum Number Of Sequences For A Flanking Position: 2
Maximum Number Of Contiguous Nonconserved Positions: 8
Minimum Length Of A Block: 10
Allowed Gap Positions: None
Use Similarity Matrices: Yes
```

```
Flank positions of the 7 selected block(s)
Flanks: [950  1068]  [1087  1210]  [1220  1274]  [1277  1287]  [1295  1334]  [1389  1403]  [1422  1440]  

New number of positions in SJ12098.aa.fasta.trans.aa_ali.fasta-gb:  383  (24% of the original 1563 positions)
```
